# Supplementary material for: The Systems Biology Research Tool: evolvable open-source software
Source: BMC Syst Biol. 2008 Jun 29;2:55. doi: 10.1186/1752-0509-2-55 (PMC2446383; doi:10.1186/1752-0509-2-55)
Supplement: Additional file 1 — SBRT Archive. An archive of the current version of the Systems Biology Research Tool. [file 1752-0509-2-55-S1.zip › sbrt-1.4.0/doc/developers_guide/api/index-files/index-3.html]

C-Index


|  |  |  |
| --- | --- | --- |
| **Overview** \| Package \| Class \| Use \| **Tree** \| **Deprecated** \| **Index** \| **Help** | |  |
| **PREV LETTER**   **NEXT LETTER** | **FRAMES**    **NO FRAMES**     **All Classes** |  |


A B C D E F G H I J K L M N O P Q R S T U V W X Y Z 

---


## **C**

**CARET** - Static variable in class sbrt.shell.text.SpecialChars: ^ **CATALYST\_DELETION\_FILE** - Static variable in class sbrt.shell.mng.fba.CatalystDeletionManager: The keyword used to denote the name of a file containing sets of names of catalysts whose effect upon deletion will be computed. **CATALYST\_FILE\_NAME** - Static variable in class sbrt.shell.mng.fba.NetworkInfoManager: The keyword used to denote teh name of the file to which all catalysts will be written. **CatalystDeletionManager** - Class in sbrt.shell.mng.fba: This class is used to manage FBA catalyst deletion experiments. **CatalystDeletionManager()** - Constructor for class sbrt.shell.mng.fba.CatalystDeletionManager: **CatalystDeletionProcess** - Class in sbrt.kernel.fba.proc: This class is used to perform catalyst deletion experiments. **CatalystDeletionProcess(RxnDeletionComputer)** - Constructor for class sbrt.kernel.fba.proc.CatalystDeletionProcess: Constructs a new catalyst deletion experiment. **CatalystFormat** - Interface in sbrt.shell.text.chem: This interface is used to represent the formats of catalysts in chemical reactions. **CatalystFormatV1** - Class in sbrt.shell.text.chem: This class is a concrete implementation of `CatalystFormat`. **CatalystFormatV1()** - Constructor for class sbrt.shell.text.chem.CatalystFormatV1: **CatalystFormatV2** - Class in sbrt.shell.text.chem: This class is a concrete implementation of `CatalystFormat`. **CatalystFormatV2()** - Constructor for class sbrt.shell.text.chem.CatalystFormatV2: **CatalystListFormat** - Interface in sbrt.shell.text.fba: This interface is used to represent the formats of lists of catalyst names. **CatalystListFormatV1** - Class in sbrt.shell.text.fba: This class is a concrete implemenation of `CatalystListFormat`. **CatalystListFormatV1(CatalyzedFluxome)** - Constructor for class sbrt.shell.text.fba.CatalystListFormatV1: Constructs a new catalyst name collection format using the provided fluxome. **catalysts()** - Method in interface sbrt.kernel.fba.CatalyzedFluxome: Returns the total number of catalysts contained in this fluxome. **catalysts()** - Method in class sbrt.kernel.fba.IrrevRxnFluxomeV1: Returns the total number of catalysts contained in this fluxome. **CatalystSetFormat** - Interface in sbrt.shell.text.fba: This interface is used to represent the formats of sets of enzyme names. **CatalystSetFormatV1** - Class in sbrt.shell.text.fba: This class is a concrete implemenation of `CatalystSetFormat`. **CatalystSetFormatV1(CatalyzedFluxome)** - Constructor for class sbrt.shell.text.fba.CatalystSetFormatV1: Constructs a new catalyst name collection format using the provided fluxome. **CatalystVerifier** - Class in sbrt.shell.text.fba: This class is used to ensure catalysts exist in a given `CatalyzedFluxome`. **CatalystVerifier(CatalyzedFluxome)** - Constructor for class sbrt.shell.text.fba.CatalystVerifier: Constructs a new catalyst name verifier for the provided fluxome. **CatalyzedFluxome** - Interface in sbrt.kernel.fba: This interface is used to represent fluxomes with catalyzed reactions. **CdHarComputer**<V> - Class in sbrt.kernel.math.geom.comp: This class is an implemenation of a coordinate-direction hit-and-run algorithm. **CdHarComputer(ConvexPolytope<V>)** - Constructor for class sbrt.kernel.math.geom.comp.CdHarComputer: Constructs a new random point generator for the provided convex polytope. **CdHarComputer.Results** - Class in sbrt.kernel.math.geom.comp: This class is used to represent the results of a random point computation. **CdHarFluxVectorComputer** - Class in sbrt.kernel.fba.comp: This class is used to compute randomly distributed vectors within flux space. **CdHarFluxVectorComputer(IrrevRxnFluxome, LinearSystemSolution<String>)** - Constructor for class sbrt.kernel.fba.comp.CdHarFluxVectorComputer: Constructs a new random flux vector computer for the provided fluxome. **CdHarFluxVectorComputer.Results** - Class in sbrt.kernel.fba.comp: This class is used to represent the results of a coordinate-direction hit-and-run flux vector computation. **CdHarFluxVectorManager** - Class in sbrt.shell.mng.fba: This class is used to manage FBA coordinate direction hit-and-run random flux vector experiments. **CdHarFluxVectorManager()** - Constructor for class sbrt.shell.mng.fba.CdHarFluxVectorManager: **CdHarFluxVectorProcess** - Class in sbrt.kernel.fba.proc: This class is used to perform coordinate direction hit-and-run flux vector experiments. **CdHarFluxVectorProcess(CdHarFluxVectorComputer)** - Constructor for class sbrt.kernel.fba.proc.CdHarFluxVectorProcess: Constructs a new coordinate direction hit-and-run flux vector experiment. **CdHarManager** - Class in sbrt.shell.mng.geom: This class is used to manage coordinate direction hit-and-run algorithms. **CdHarManager()** - Constructor for class sbrt.shell.mng.geom.CdHarManager: **CdHarProcess**<V> - Class in sbrt.kernel.math.geom.proc: This class is used to generate random, uniformly distributed points from the interior of a convex polytope. **CdHarProcess(ConvexPolytope<V>)** - Constructor for class sbrt.kernel.math.geom.proc.CdHarProcess: Constructs a new random point generator process for the provided polytope. **changedVariables()** - Method in class sbrt.kernel.math.geom.comp.CdHarComputer.Results: Returns the number of variables whose values are different than they were in the previous point. **check(ManagerInput<String, String>)** - Method in class sbrt.shell.mng.AbstractProcessManager: Throws an ApplicationException if this `ProcessManager` cannot understand the provided input. **check(ManagerInput<K, V>)** - Method in interface sbrt.shell.mng.ProcessManager: Throws an `ApplicationException` if this `ProcessManager` cannot understand the provided input. **checkCatalyst(String)** - Method in class sbrt.kernel.fba.IrrevRxnFluxomeV1: Ensures the provided string is a valid catalyst. **checkChemSpecies(String)** - Method in class sbrt.kernel.fba.comp.FbaOptComputer: Ensures the provided string is the name of a chemical species in the fluxome used by this optimization computer. **checkColExpr(MathExpr<?>)** - Method in class sbrt.kernel.fba.comp.FbaOptComputer: Ensures all the variables in the provided expression are valid column variables. **checkColumnValue(Object, Double)** - Method in class sbrt.kernel.fba.comp.FbaOptComputer: Ensures the provided numerical value does not violate the defined constraint of the provided column variable. **checkColumnValue(Object, double)** - Method in class sbrt.kernel.fba.comp.FbaOptComputer: Ensures the provided numerical value does not violate the defined constraint of the provided column variable. **checkColumnVariable(Object)** - Method in class sbrt.kernel.fba.comp.FbaOptComputer: Ensures the provided object is column variable. **checkColumnVariables(Collection<?>)** - Method in class sbrt.kernel.fba.comp.FbaOptComputer: Ensures each element of the provided collection is column variable. **checkConstant(double)** - Static method in class sbrt.kernel.math.AbstractPolynomial: Ensures the provided constant is not infinite and not NaN. **checkFile(String)** - Static method in class sbrt.shell.io.FileLineIterator: Ensures the provided string is the name of a valid file. **checkFluxConstraint(Interval, String, double)** - Method in class sbrt.kernel.fba.comp.AbstractCvComputer: Ensures the flux constraint is valid for the fluxome provided. **checkFluxConstraint(Interval, String, double)** - Method in class sbrt.kernel.fba.comp.FbaOptComputer: Ensures the provided constraint is a valid flux constraint in the fluxome used by this optimization computer. **checkForConflict(FluxConstraints, Set<String>)** - Static method in class sbrt.kernel.fba.proc.CvRxnDeletionProcess: Ensures that the provided flux constraints and set of reaction names do not conflict. **checkNumber(double)** - Method in class sbrt.kernel.fba.comp.AbstractCvComputer: Ensures the provided number is not `NaN` or infinite. **checkNumber(double)** - Method in class sbrt.kernel.fba.comp.FbaOptComputer: Ensures the provided number is finite and not NaN. **checkRowVariable(Object)** - Method in class sbrt.kernel.fba.comp.FbaOptComputer: Ensures the provided object is row variable. **checkRxnExpr(MathExpr<String>)** - Method in class sbrt.kernel.fba.comp.AbstractCvComputer: Ensures the expression is valid for the fluxome provided. **checkRxnExpr(MathExpr<String>)** - Method in class sbrt.kernel.fba.comp.FbaOptComputer: Ensures all the variables in the provided expression are reaction names. **checkRxnExprConstraint(Interval)** - Method in class sbrt.kernel.fba.comp.AbstractCvComputer: Ensures the expression constraint is valid for the fluxome provided. **checkRxnExprConstraint(Interval)** - Method in class sbrt.kernel.fba.comp.FbaOptComputer: Ensures the provided constraint is valid for reaction name-expressions for the fluxome used by this optimization computer. **checkRxnName(String)** - Method in class sbrt.kernel.fba.comp.AbstractCvComputer: Ensures the reaction name is valid for the fluxome provided. **checkRxnName(String)** - Method in class sbrt.kernel.fba.comp.FbaOptComputer: Ensures the provided string is the name of a reaction in the fluxome used by this optimization computer. **checkRxnName(String)** - Method in interface sbrt.kernel.fba.IrrevRxnFluxome: Ensures the provided string is a valid reaction name. **checkRxnName(String)** - Method in class sbrt.kernel.fba.IrrevRxnFluxomeV1: Ensures the provided string is a valid reaction name. **checkRxnNames(Collection<String>)** - Method in class sbrt.kernel.fba.comp.AbstractCvComputer: Ensures the reaction names are valid for the fluxome provided. **checkRxnNames(Collection<String>)** - Method in class sbrt.kernel.fba.comp.FbaOptComputer: Ensures each element of the provided collection is reaction name. **checkRxnNames(Collection<String>)** - Method in interface sbrt.kernel.fba.IrrevRxnFluxome: Ensures each element of the provided collection is a valid reaction name. **checkRxnNames(Collection<String>)** - Method in class sbrt.kernel.fba.IrrevRxnFluxomeV1: Ensures each element of the provided collection is a valid reaction name. **checkSolutionVector(Map<?, Double>)** - Method in class sbrt.kernel.fba.comp.FbaOptComputer: Ensures the provided solution vector does not violate the defined constraints of the system. **checkState()** - Method in class sbrt.kernel.io.AbstractResultsManager: Ensures `close()` has not already been called. **CHEM\_SPECIES\_FILE\_NAME** - Static variable in class sbrt.shell.mng.fba.NetworkInfoManager: The keyword used to denote the name of the file to which the chemical species of a fluxome will be written. **ChemRxn** - Interface in sbrt.kernel.fba: This interface is used to represent simple chemical reactions. **ChemSpeciesFormat** - Interface in sbrt.shell.text.chem: This interface is used to represent the formats of chemical species. **ChemSpeciesFormatV1** - Class in sbrt.shell.text.chem: This class is a concrete implemenation of `ChemSpeciesFormat`. **ChemSpeciesFormatV1()** - Constructor for class sbrt.shell.text.chem.ChemSpeciesFormatV1: **ChemSpeciesVerifier** - Class in sbrt.shell.text.fba: This class is used to ensure chemical species exist in a given `Fluxome`. **ChemSpeciesVerifier(Fluxome)** - Constructor for class sbrt.shell.text.fba.ChemSpeciesVerifier: Constructs a new chemical species verifier for the provided fluxome. **clear()** - Method in class sbrt.ext.cplex.CplexSolver: Clears all information contained in this program solver. **clear()** - Method in class sbrt.ext.glpk.GlpkSolver: Clears all information contained in this program solver. **clear()** - Method in interface sbrt.kernel.math.optimization.ProgramSolver: Clears all information contained in this program solver. **clear()** - Method in class sbrt.kernel.util.UnmodifiableCollection: See Collection.clear(). **clear()** - Method in class sbrt.kernel.util.UnmodifiableList: See List.clear(). **clear()** - Method in class sbrt.kernel.util.UnmodifiableMap: See Map.clear(). **clear()** - Method in class sbrt.kernel.util.UnmodifiableSet: See Set.clear(). **close()** - Method in class sbrt.ext.mma.algebra.MathematicaSolver: Closes the link to the Mathematica kernel. **close()** - Method in class sbrt.kernel.io.AbstractResultsManager: Closes this results manager. **close()** - Method in interface sbrt.kernel.io.ResultsManager: Closes this results manager so that no further results can be added. **close()** - Method in class sbrt.shell.io.AbstractMapInputFile: Closes this file. **close()** - Method in class sbrt.shell.io.AbstractMapOutputFile: Closes this file. **close()** - Method in class sbrt.shell.io.fba.ColumnOrFbaOptOutputFile: Closes this file. **close()** - Method in class sbrt.shell.io.fba.FluxCapInputFile: Closes this file. **close()** - Method in class sbrt.shell.io.fba.FluxIntervalInputFile: Closes this file. **close()** - Method in class sbrt.shell.io.fba.RowOrFbaOptOutputFile: Closes this file. **close()** - Method in class sbrt.shell.io.fba.SerFbaOptOutputFile: Closes this file. **close()** - Method in class sbrt.shell.io.MultiOutputFiles: Closes this results manager so that no further results can be added. **close()** - Method in interface sbrt.shell.io.SbrtFile: Closes this file. **close()** - Method in class sbrt.shell.io.SerializedFileIterator: Closes this file iterator. **close()** - Method in class sbrt.shell.io.SerializedInputFile: Closes this file. **close()** - Method in class sbrt.shell.io.SerializedOutputFile: Closes this file. **close()** - Method in class sbrt.shell.io.TextFileLineIterator: Closes this file iterator. **close()** - Method in class sbrt.shell.io.TextInputFile: Closes this file. **close()** - Method in class sbrt.shell.io.TextOutputFile: Closes this file. **closeFile(SbrtFile, PrintWriter)** - Static method in class sbrt.shell.mng.AbstractProcessManager: Closes the provided results manager and prints a descriptive message to the provided print writer. **closeRm(ResultsManager, PrintWriter)** - Static method in class sbrt.shell.mng.AbstractProcessManager: Closes the provided file and prints a descriptive message to the provided print writer. **CoeffFormat** - Interface in sbrt.shell.text.math: This interface is used to represent the format of coefficients of mathematical expressions. **CoeffFormatV1** - Class in sbrt.shell.text.math: This class is a concrete implementation of `CoeffFormat`. **CoeffFormatV1()** - Constructor for class sbrt.shell.text.math.CoeffFormatV1: Constructs a new coefficient format object. **CoeffFormatV2** - Class in sbrt.shell.text.math: This class is a concrete implementation of `CoeffFormat`. **CoeffFormatV2()** - Constructor for class sbrt.shell.text.math.CoeffFormatV2: Constructs a new coefficient format object. **CofoofSource** - Class in sbrt.shell.io.fba: This class is used to provide a source of column-oriented FBA optimization output files. **CofoofSource(Iterator<String>, FileFormat, Set<String>, FbaOptFileLineFormat)** - Constructor for class sbrt.shell.io.fba.CofoofSource: Constructs a new source of column-oriented FBA optimization output files. **CofoofSource(Iterator<String>, String, FileFormat, Set<String>, FbaOptFileLineFormat)** - Constructor for class sbrt.shell.io.fba.CofoofSource: Constructs a new source of column-oriented FBA optimization output files. **CollectionFormat**<E> - Interface in sbrt.shell.text: This interface is used to represent collection formats. **COLON** - Static variable in class sbrt.shell.text.SpecialChars: : **ColonMapEntryFormatV1**<K,V> - Class in sbrt.shell.text: This class is a concrete implementation of `MapEntryFormat`. **ColonMapEntryFormatV1(SimpleFormat<K>, SimpleFormat<V>)** - Constructor for class sbrt.shell.text.ColonMapEntryFormatV1: Constructs a new map entry format using the provided key and value formats. **ColonMapFormatV1**<K,V> - Class in sbrt.shell.text: This class is a concrete implementation of `MapFormat`. **ColonMapFormatV1(SimpleFormat<K>, SimpleFormat<V>)** - Constructor for class sbrt.shell.text.ColonMapFormatV1: Constructs a new map format using the provided key and value formats. **ColumnOrFbaOptOutputFile** - Class in sbrt.shell.io.fba: This class is used to write column-oriented output files containing FBA optimization data. **ColumnOrFbaOptOutputFile(String, FileFormat, Set<String>, FbaOptFileLineFormat)** - Constructor for class sbrt.shell.io.fba.ColumnOrFbaOptOutputFile: Constructs a new column-oriented FBA optimization output file. **columns()** - Method in class sbrt.kernel.math.algebra.HashMatrix: Returns the number of columns in this matrix. **columns()** - Method in interface sbrt.kernel.math.algebra.Matrix: Returns the number of columns in this matrix. **columns()** - Method in class sbrt.kernel.math.algebra.MatrixWrap: Returns the number of columns in this matrix. **columns()** - Method in class sbrt.kernel.math.algebra.RowOrTreeMatrix: Returns the number of columns in this matrix. **columns()** - Method in class sbrt.kernel.math.algebra.TreeMatrix: Returns the number of columns in this matrix. **ComiFile**<K,V> - Class in sbrt.shell.io: This class is used to read column-oriented map input files. **ComiFile(String, FileFormat, Set<? extends K>, MapFormat<K, V>)** - Constructor for class sbrt.shell.io.ComiFile: Constructs a new row-oriented map input file from the provided arguments. **ComiFile(String, FileFormat, MapFormat<K, V>)** - Constructor for class sbrt.shell.io.ComiFile: Constructs a new row-oriented map input file from the provided arguments. **ComiFile(String, MapFormat<K, V>)** - Constructor for class sbrt.shell.io.ComiFile: Constructs a new row-oriented map input file from the provided arguments. **ComiFileSource**<K,V> - Class in sbrt.shell.io: This class is used to provided a source of column-oriented map input files. **ComiFileSource(Iterator<String>, FileFormat, Set<? extends K>, MapFormat<K, V>)** - Constructor for class sbrt.shell.io.ComiFileSource: Constructs a new source of column-oriented map input files. **ComiFileSource(Iterator<String>, Set<? extends K>, MapFormat<K, V>)** - Constructor for class sbrt.shell.io.ComiFileSource: Constructs a new source of column-oriented map input files. **ComiFileSource(Iterator<String>, FileFormat, MapFormat<K, V>)** - Constructor for class sbrt.shell.io.ComiFileSource: Constructs a new source of column-oriented map input files. **COMMA** - Static variable in class sbrt.shell.text.SpecialChars: , **ComoFile**<K,V> - Class in sbrt.shell.io: This class is used to write maps to text files in a column-oriented way. **ComoFile(String, FileFormat, Set<? extends K>, MapFormat<K, V>)** - Constructor for class sbrt.shell.io.ComoFile: Constructs a new column-oriented map output file from the provided arguments. **ComoFile(String, FileFormat, String, MapFormat<K, V>)** - Constructor for class sbrt.shell.io.ComoFile: Constructs a new column-oriented vector output file from the provided arguments. **ComoFile(String, Set<? extends K>, MapFormat<K, V>)** - Constructor for class sbrt.shell.io.ComoFile: Constructs a new text-based column-oriented map output file from the provided arguments. **ComoFileSource**<K,V> - Class in sbrt.shell.io: This class is used to provided a source of column-oriented map output files. **ComoFileSource(Iterator<String>, FileFormat, Set<? extends K>, MapFormat<K, V>)** - Constructor for class sbrt.shell.io.ComoFileSource: Constructs a new source of column-oriented map output files. **ComoFileSource(Iterator<String>, FileFormat, String, MapFormat<K, V>)** - Constructor for class sbrt.shell.io.ComoFileSource: Constructs a new source of column-oriented map output files. **ComoFileSource(Iterator<String>, FileFormat, MapFormat<K, V>)** - Constructor for class sbrt.shell.io.ComoFileSource: Constructs a new source of column-oriented map output files. **complete()** - Method in class sbrt.kernel.util.PercentProgressMonitor: Sets the internal counter equal to the provided total. **COMPLETED** - Static variable in class sbrt.shell.mng.AbstractProcessManager: The prefix used to denote the number of completed operations. **ConflictingInputException** - Exception in sbrt.kernel.io: This class is used to indicate conflicting inputs. **ConflictingInputException(List<?>)** - Constructor for exception sbrt.kernel.io.ConflictingInputException: Constructs a new conflicting input exception. **CONSTRAINT\_TOLERANCE** - Static variable in class sbrt.shell.mng.fba.FbaManager: The keyword used to denote a constraint tolerance. **CONSTRAINT\_TOLERANCE** - Static variable in class sbrt.shell.mng.geom.CdHarManager: The keyword used to denote the constraint tolerance. **CONSTRAINTS\_FILE** - Static variable in class sbrt.shell.mng.fba.FbaOptManager: The keyword used to denote a constraints file. **ConstraintsFileLineFormat**<E> - Interface in sbrt.shell.text.fba: This interface is used to represent formats for flux constraints files. **ConstraintsFileLineFormatV1** - Class in sbrt.shell.text.fba: This class is a concrete implemenation of `ConstraintsFileLineFormat`. **ConstraintsFileLineFormatV1(Fluxome)** - Constructor for class sbrt.shell.text.fba.ConstraintsFileLineFormatV1: Constructs a new contraints file line format. **ConstraintsFileLineFormatV2** - Class in sbrt.shell.text.fba: This class is a concrete implemenation of `ConstraintsFileLineFormat`. **ConstraintsFileLineFormatV2(Fluxome)** - Constructor for class sbrt.shell.text.fba.ConstraintsFileLineFormatV2: Constructs a new contraints file line format. **ConstraintViolationException** - Exception in sbrt.kernel.math: This class is used to indicate a constraint violation. **ConstraintViolationException(Object, Interval, double)** - Constructor for exception sbrt.kernel.math.ConstraintViolationException: Constructs a new constraint violation exception. **ConstraintViolationException(Object, Interval, Number)** - Constructor for exception sbrt.kernel.math.ConstraintViolationException: Constructs a new constraint violation exception. **contains(Object)** - Method in class sbrt.kernel.math.graph\_theory.Path: Returns true if this path contains the specified node. **contains(E)** - Method in class sbrt.kernel.util.BooleanRelation: **contains(Object)** - Method in class sbrt.kernel.util.UnmodifiableCollection: See Collection.contains(Object). **contains(Object)** - Method in class sbrt.kernel.util.UnmodifiableList: See List.contains(Object). **contains(Object)** - Method in class sbrt.kernel.util.UnmodifiableSet: See Set.contains(Object). **containsAll(Collection<?>)** - Method in class sbrt.kernel.util.UnmodifiableCollection: See Collection.containsAll(Collection). **containsAll(Collection<?>)** - Method in class sbrt.kernel.util.UnmodifiableList: See List.containsAll(Collection). **containsAll(Collection<?>)** - Method in class sbrt.kernel.util.UnmodifiableSet: See Set.containsAll(Collection). **containsKey(Object)** - Method in class sbrt.kernel.util.UnmodifiableMap: See Map.containsKey(Object). **containsValue(Object)** - Method in class sbrt.kernel.util.UnmodifiableMap: See Map.containsValue(Object). **convertConstraints(Map<LinearComb<String>, Interval>)** - Static method in class sbrt.shell.io.fba.FluxIntervalInputFile: Converts the provided intervals into a `FluxConstraints` object. **convertVariables(Set<String>)** - Static method in class sbrt.shell.io.fba.FluxIntervalInputFile: Converts a set of reaction names to a set of single-term linear combinations. **ConvexPolytope**<V> - Interface in sbrt.kernel.math.geom: This interface is used to represent convex polytopes that are represented as the intersection of a finite set of half-spaces. **corr(Map<? extends V, Double>, Map<? extends V, Double>)** - Method in class sbrt.ext.R.stat.RCorrelator: Computes and returns the correlation statistic of the provided x and y values. **corr(Double[], Double[])** - Method in class sbrt.ext.R.stat.RCorrelator: Computes and returns the correlation results for the provided values. **corr(Collection<Double>, Collection<Double>)** - Method in class sbrt.ext.R.stat.RCorrelator: Computes and returns the correlation results for the provided values. **corr(Map<? extends V, Double>, Map<? extends V, Double>)** - Method in interface sbrt.kernel.math.stat.comp.Correlator: Computes and returns the correlation statistic of the provided x and y values. **corr(Collection<Double>, Collection<Double>)** - Method in interface sbrt.kernel.math.stat.comp.Correlator: Computes and returns the correlation statistic of the provided x and y values. **corr(Double[], Double[])** - Method in interface sbrt.kernel.math.stat.comp.Correlator: Computes and returns the correlation statistic of the provided x and y values. **corr(Map<? extends V, Double>, Map<? extends V, Double>)** - Method in class sbrt.kernel.math.stat.comp.KendallTau: Computes and returns the correlation results for the provided values. **corr(Collection<Double>, Collection<Double>)** - Method in class sbrt.kernel.math.stat.comp.KendallTau: Computes and returns the correlation results for the provided values. **corr(Double[], Double[])** - Method in class sbrt.kernel.math.stat.comp.KendallTau: Computes and returns the correlation results for the provided values. **corr(double[], double[])** - Method in class sbrt.kernel.math.stat.comp.KendallTau: Computes and returns the correlation results for the provided values. **CORR\_COEFF** - Static variable in class sbrt.shell.mng.stat.CorrelationManager: The keyword used to denote the type of correlation coefficient to be computed. **CorrelationAnalysis**<V,T extends CorrResult> - Class in sbrt.kernel.math.stat.proc: This class is used to perform correlation experiments. **CorrelationAnalysis(Correlator<V, T>)** - Constructor for class sbrt.kernel.math.stat.proc.CorrelationAnalysis: Constructs a new correlation experiment. **CorrelationManager** - Class in sbrt.shell.mng.stat: This class is used to manage correlation processes. **CorrelationManager()** - Constructor for class sbrt.shell.mng.stat.CorrelationManager: **Correlator**<V,T extends CorrResult> - Interface in sbrt.kernel.math.stat.comp: This interface is used to represent correlation statistic computers. **CorrResFormatterV1** - Class in sbrt.shell.text.math: This class is a concrete implementation of `CorrResultsFormatter`. **CorrResFormatterV1()** - Constructor for class sbrt.shell.text.math.CorrResFormatterV1: Constructs a new correlation results formatter. **CorrResult** - Interface in sbrt.kernel.math.stat.comp: This interface is used to represent objects that store the results of a correlation computation. **CorrResultsFormatter**<T> - Interface in sbrt.shell.text.math: This interface is used to format the results of a correlation computation. **CPLEX** - Static variable in class sbrt.shell.ProgramSolverFactory: The string used to denote the CPLEX program solver from ILOG. **CplexSolver**<R,C> - Class in sbrt.ext.cplex: This class is a wrapper for the CPLEX package from ILOG. **CplexSolver()** - Constructor for class sbrt.ext.cplex.CplexSolver: Constructs a new CPLEX program solver. **createAndShowWindow()** - Method in class sbrt.shell.gui.SbrtGui: Creates and shows the SBRT's GUI. **createAndShowWindow()** - Method in class sbrt.shell.gui.TextPaneGui: Creates and shows the SBRT's GUI. **CV\_FILE** - Static variable in class sbrt.shell.mng.fba.IntervalVariationManager: The keyword used to denote a constraint variation input file. **CvCatalystDeletionManager** - Class in sbrt.shell.mng.fba: This class is used to manage FBA constraint variation-catalyst deletion experiments. **CvCatalystDeletionManager()** - Constructor for class sbrt.shell.mng.fba.CvCatalystDeletionManager: **CvCatalystDeletionProcess** - Class in sbrt.kernel.fba.proc: This class is used to perform constraint-variation catalyst deletion experiments. **CvCatalystDeletionProcess(RxnDeletionComputer)** - Constructor for class sbrt.kernel.fba.proc.CvCatalystDeletionProcess: Constructs a new constraint-variation catalyst deletion experiment. **CvFbaOptimizationExperiment** - Class in sbrt.kernel.fba.proc: This class is used to perform constraint-variation FBA optimization experiments. **CvFbaOptimizationExperiment(FbaOptComputer)** - Constructor for class sbrt.kernel.fba.proc.CvFbaOptimizationExperiment: Constructs a new constraint-variation FBA optimization experiment. **CvFbaOptManager** - Class in sbrt.shell.mng.fba: This class is used to manage constraint variation-FBA optimization processes. **CvFbaOptManager()** - Constructor for class sbrt.shell.mng.fba.CvFbaOptManager: **CvFbaOptProcess**<R> - Class in sbrt.kernel.fba.proc: This class is used to represent constraint-variation Flux Balance Analysis experiments. **CvFbaOptProcess()** - Constructor for class sbrt.kernel.fba.proc.CvFbaOptProcess: **CvFluxIntervalProcess** - Class in sbrt.kernel.fba.proc: This class is used to perform constraint variation flux interval experiments. **CvFluxIntervalProcess(FluxIntervalComputer)** - Constructor for class sbrt.kernel.fba.proc.CvFluxIntervalProcess: Constructs a new constraint variation flux interval experiment. **CvMsFluxIntervalManager** - Class in sbrt.shell.mng.fba: This class is used to manage constraint variation-Mahadevan-Schilling flux interval experiments. **CvMsFluxIntervalManager()** - Constructor for class sbrt.shell.mng.fba.CvMsFluxIntervalManager: **CvObjectiveFunctionManager** - Class in sbrt.shell.mng.fba: This class is used to manage FBA constraint variation-objective function experiments. **CvObjectiveFunctionManager()** - Constructor for class sbrt.shell.mng.fba.CvObjectiveFunctionManager: **CvObjectiveFunctionProcess** - Class in sbrt.kernel.fba.proc: This class is used to perform constraint-variation objective function experiments. **CvObjectiveFunctionProcess(FbaOptComputer)** - Constructor for class sbrt.kernel.fba.proc.CvObjectiveFunctionProcess: Constructs a new constraint-variation objective function experiment. **CvRevRxnFluxIntervalManager** - Class in sbrt.shell.mng.fba: This class is used to manage constraint variation-constrained reverse reaction flux interval experiments. **CvRevRxnFluxIntervalManager()** - Constructor for class sbrt.shell.mng.fba.CvRevRxnFluxIntervalManager: **CvRxnDeletionManager** - Class in sbrt.shell.mng.fba: This class is used to manage FBA constraint variation-reaction deletion experiments. **CvRxnDeletionManager()** - Constructor for class sbrt.shell.mng.fba.CvRxnDeletionManager: **CvRxnDeletionProcess** - Class in sbrt.kernel.fba.proc: This class is used to perform constraint-variation reaction deletion experiments. **CvRxnDeletionProcess(RxnDeletionComputer)** - Constructor for class sbrt.kernel.fba.proc.CvRxnDeletionProcess: Constructs a new constraint-variation reaction deletion experiment. **CvSimpleFluxIntervalManager** - Class in sbrt.shell.mng.fba: This class is used to manage constraint variation-simple flux interval experiments. **CvSimpleFluxIntervalManager()** - Constructor for class sbrt.shell.mng.fba.CvSimpleFluxIntervalManager: **CYCLE\_FILE** - Static variable in class sbrt.shell.mng.fba.FluxCapManager: The keyword used to denote the steady state cycle input file. **CycleComputer** - Interface in sbrt.kernel.fba.comp: This interface is used to represent algorithms for identifying steady state cycles (Type III extreme pathways) in stoichiometric networks. **CycleIdManager** - Class in sbrt.shell.mng.graph\_theory: This class is used to manage simple cycle identification experiments. **CycleIdManager()** - Constructor for class sbrt.shell.mng.graph\_theory.CycleIdManager: **CycleIdProcess** - Class in sbrt.kernel.fba.proc: This class is used to execute steady state cycle identification processes. **CycleIdProcess(IrrevRxnFluxome, CycleComputer)** - Constructor for class sbrt.kernel.fba.proc.CycleIdProcess: Constructs a new steady state cycle identification process. **CycleIdProcess**<N> - Class in sbrt.kernel.math.graph\_theory.proc: This class is used to identify all the simple cycles in a directed graph. **CycleIdProcess()** - Constructor for class sbrt.kernel.math.graph\_theory.proc.CycleIdProcess: Constructs a new cycle-identification experiment.

---


|  |  |  |
| --- | --- | --- |
| **Overview** \| Package \| Class \| Use \| **Tree** \| **Deprecated** \| **Index** \| **Help** | |  |
| **PREV LETTER**   **NEXT LETTER** | **FRAMES**    **NO FRAMES**     **All Classes** |  |


A B C D E F G H I J K L M N O P Q R S T U V W X Y Z 

---
